# Supplementary material for: EnzML: multi-label prediction of enzyme classes using InterPro signatures
Source: BMC Bioinformatics. 2012 Apr 25;13:61. doi: 10.1186/1471-2105-13-61 (PMC3483700; doi:10.1186/1471-2105-13-61)
Supplement: Addtional file 5 — The Java code to format the data files, evaluate and predict. The file enzml_java_code.tar.gz contains the Java code used to format database data to ARFF and XML formats, to execute cross and train-test (jackknife) evaluations and to record evaluation results to database. More information is included in the readme.txt file and the Javadoc files. The code can be used with a MySQL database. To use a different database software, other JDBC drivers might be required. [file 1471-2105-13-61-S5.gz › java_code/utils/doc/uk/ac/ed/inf/utils/database/package-use.html]

Uses of Package uk.ac.ed.inf.utils.database


---


|  |  |  |
| --- | --- | --- |
| **Overview** \| **Package** \| Class \| **Use** \| **Tree** \| **Deprecated** \| **Index** \| **Help** | |  |
| PREV   NEXT | **FRAMES**    **NO FRAMES**     **All Classes** |  |


---


## **Uses of Package uk.ac.ed.inf.utils.database**

| Packages that use uk.ac.ed.inf.utils.database | |
| --- | --- |
| **test.database** |  |
| **uk.ac.ed.inf.utils.database** |  |
| **uk.ac.ed.inf.utils.setutils** |  |

Classes in uk.ac.ed.inf.utils.database used by test.database

****DbConn****
  
          A DbConnectionProperties object contains the properties needed to connect to
a database through JDBC.

****DbCreator****
  
          Class

****DbManager****
  
          A database manager provides object to create a database, write and read from
it.

****DbReader****
  
          Class to read from a database.

****DbWriter****
  
          To write to a database table

****Table****
  
          An SQL database table definition (table name + columns)

****TableManager****
  
          Class to create/read/write to a database table.

****TableRow****
  
          Contains the data for a generic row (a list of [table column, value] entries)
to be written to a database table.

Classes in uk.ac.ed.inf.utils.database used by uk.ac.ed.inf.utils.database

****DbConn****
  
          A DbConnectionProperties object contains the properties needed to connect to
a database through JDBC.

****DbCreator****
  
          Class

****DbManaged****
  
          Managed is a class which has a `DbManager` (an object that coordinates
its database activities)

****DbManager****
  
          A database manager provides object to create a database, write and read from
it.

****DbReader****
  
          Class to read from a database.

****DbWriter****
  
          To write to a database table

****Manager****
  
          A manager manages: for example, for a database object, the `DbManager`
manages a `DbCreator` to create the tables, `DbReader` to read
data from and `DbWriter` to write data to the database.

****Table****
  
          An SQL database table definition (table name + columns)

****TableColumn****
  
          A column object contains all the information to create, fill and delete a
database table column

****TableCreator****
  
          Class to create a table object in the database.

****TableManaged****
  
          TableManaged is a class which has a `TableManager` (an object that
coordinates its table activities)

****TableManager****
  
          Class to create/read/write to a database table.

****TableReader****
  
          Class

****TableRow****
  
          Contains the data for a generic row (a list of [table column, value] entries)
to be written to a database table.

****TableWriter****
  
          A TableWriter object contains all the information to create, fill and delete
a database table.

Classes in uk.ac.ed.inf.utils.database used by uk.ac.ed.inf.utils.setutils

****DbManager****
  
          A database manager provides object to create a database, write and read from
it.

---


|  |  |  |
| --- | --- | --- |
| **Overview** \| **Package** \| Class \| **Use** \| **Tree** \| **Deprecated** \| **Index** \| **Help** | |  |
| PREV   NEXT | **FRAMES**    **NO FRAMES**     **All Classes** |  |


---
